# Acceptability of Technologies to Support Early Dementia Detection: Qualitative Study With the Boston University Alzheimer’s Disease Center Cohort

**DOI:** 10.2196/84004

**Published:** 2026-05-29

**Authors:** Sarah Wilson, Emily Beswick, Zachary Popp, Salman Rahman, Sharandeep Bhogal, Tim Whitfield, Spencer Low, Raiyan Khan, Clare Tolley, Zuzana Walker, Rhoda Au, Sarah P Slight

**Affiliations:** 1 School of Pharmacy, Newcastle University, King George VI Building, King’s Road, Newcastle Upon Tyne, NE1 7RU, United Kingdom, 44 0191 208 2335; 2 Trinity College Dublin, Dublin, Ireland; 3 Alzheimer’s Disease Research Centre, Boston University, Boston, MA, United States; 4 University College London, London, United Kingdom

**Keywords:** digital technology, wearables, early detection, mild cognitive impairment, MCI, aging, qualitative research

## Abstract

**Background:**

Dementia is on the rise globally due to increasing life expectancies and population growth. Digital technologies may help detect early signs, enabling timely interventions to slow or reverse cognitive decline. However, to support the successful implementation of these digital technologies into health care settings, they must be acceptable to target users. Older adults and those with mild cognitive impairment (MCI) are at risk of developing dementia in later life and need to be able to use these technologies in order for this intervention to be approved and implemented in clinical practice.

**Objective:**

This study explored the perspectives of older adults and those living with a clinical diagnosis of MCI on the acceptability of using various digital technologies that have the potential to support early dementia detection.

**Methods:**

Participants were recruited from Boston University’s Alzheimer’s Disease Research Center. Participants selected at least 2 technologies from 9 different wearables and software to use for 2 weeks, at 3-month intervals, over a total duration of 2 years. A subgroup of self-selecting participants was interviewed after the first 2 weeks of use to gather initial perspectives regarding the acceptability of using the digital technologies. An inductive framework thematic analysis approach was used, assisted by NVivo (version 14.23.2; QSR International).

**Results:**

In total, 13 individuals living with a clinical diagnosis of MCI and 11 adults aged 65 years and older were interviewed. Our analysis identified five key themes: (1) gamification, (2) wearability, (3) user guidance, (4) burden of use, and (5) usefulness. Gamified apps were generally liked, although users with little experience of digital games needed time to adjust. Wearables resembling everyday accessories (eg, watches) were preferred, but complaints about tight or uncomfortable straps were frequently reported. Clear instructions were critical to support correct use, but many participants would have liked more troubleshooting support when technical issues arose. The use of 5 or more devices led to a high burden, especially when devices had practicality issues such as not being waterproof. Devices offering personal feedback were perceived as useful to satisfy personal interests, though some questioned their usefulness within health care. Participants raised concerns about losing valued personal interactions with health care professionals and questioned how their existing health conditions and treatment for such conditions may affect the validity of the data collected by the devices.

**Conclusions:**

These findings can guide researchers in choosing appropriate devices and minimizing burden. Future work should explore the views of those experiencing digital exclusion to ensure equitable access to dementia-detection technologies.

## Introduction

Over 57 million people lived with dementia in 2021 [1]. The number of new cases has been estimated to increase by nearly 10 million annually [1]. The most common dementia-causing disease is Alzheimer disease, contributing to 60%‐70% of cases [1]. Neuropathological features of dementia-causing diseases start to occur up to 2 decades before the appearance of symptoms that are clinically detectable using current diagnostic techniques [2]. These diagnostic techniques often include a combination of pen-and-paper cognitive assessments to evaluate cognitive function, such as the Montreal Cognitive Assessment [3], neuroimaging techniques to detect potential structural changes associated with dementia, and health care professionals’ judgment [4,5]. Researchers have explored the potential of cerebrospinal fluid or positron emission tomography imaging to detect biomarkers associated with the early signs of dementia-causing diseases [6]. However, these methods are invasive to patients and are not feasible to implement on a wide scale due to the considerable burden on health care resources (eg, financial cost, staff training or time, and scanning facilities) [7-9].

Over recent years, there has been significant interest in the use of digital technologies to support the early detection of dementia-causing diseases in a cost-effective, less invasive manner [9]. Digital technologies, such as wearable devices and software, have the potential to facilitate remote monitoring of individuals’ cognitive abilities and function [9,10]. These different technologies can be used for Alzheimer disease and related dementia assessment, covering diverse populations such as Lewy body, vascular dementias, frontotemporal dementias, and all severities of Alzheimer disease [11]. The successful implementation of such technologies into health care has been suggested to rely heavily on end users’ acceptance of using these digital technologies [12]. International public health agencies, such as the European Medicines Agency, the US Food and Drug Administration, and the National Institute for Health and Care Excellence, also require evidence of user involvement in the design and development of digital technology to ensure that patients understand how to use the digital technology and accept the design before the technology can be approved and implemented into clinical practice [13-15]. Those at risk of developing dementia later in life are likely to be the end users of such technologies. This includes older adults (aged 65 years and older) and those living with a clinical diagnosis of mild cognitive impairment (MCI) [16,17]. MCI is characterized by noticeable changes in memory or other cognitive abilities, such as the loss of language skills or visual or spatial perception, that are greater than what is expected with normal aging but not severe enough to interfere with daily life [18]. Although not everyone with a diagnosis of MCI progresses to dementia [19], those with an MCI diagnosis have a 5-fold increased risk of developing dementia later in life [17]. Therefore, to help identify acceptable digital technologies that could potentially support the early detection of dementia-causing diseases, we conducted a qualitative study to explore the perspectives of older adults and those living with MCI on the acceptability of using a range of different digital technologies.

## Methods

### Study Design

This study was part of the Early Detection of Neurodegenerative Diseases (EDoN) initiative, which aimed to develop a “digital toolkit” that contained technologies with the potential to detect early signs of dementia-causing diseases [9]. To support the development of the EDoN digital toolkit, we conducted semistructured interviews with potential end users (older adults and those living with MCI) to explore factors affecting concurrent acceptability (the extent to which an individual receiving an intervention finds it appropriate while participating [20]) of different digital technologies.

### Participant Recruitment and Eligibility

Participants were recruited through an EDoN-affiliated research cohort at Boston University-Alzheimer’s Disease Research Centre (BU-ADRC). The BU-ADRC is a National Institute of Health–funded center that provides resources to support Alzheimer disease research, including a network of individuals without cognitive decline, MCI, and dementia, all older than 50 years of age and willing to take part in research [21]. Individuals were eligible to take part if they owned a smartphone, had access to the internet at home, and were fluent in the English language.

### Procedure

Participants were informed of the 9 different commercially available digital technologies to choose from, what the technologies did (ie, modalities they measured), and how to use them. Participants then chose a minimum of 2 technologies to use over the course of 3 months (Table 1). These digital technologies were selected by EDoN and BU-ADRC for the study, as they each measure different modalities associated with the early signs of dementia-causing diseases and thus have the potential to support early detection. These technologies included 3 gamified active smartphone apps (ie, involve an element of gameplay, such as following to achieve a goal), 2 passive apps (smartphone and computer), and 4 wearable technologies (2 wrist-worn activity monitors, a smart ring, and an electroencephalogram headband; the modalities each digital technology measured are presented in Table 1). A member of the research team (ZP and SR), who had received training on how to set up and use the technologies by the technology companies and developers, provided each participant with their selected digital technologies and supported the initial onboarding process during an in-person visit. The onboarding process involved activities such as helping participants download the relevant apps, create accounts, and pair the wearable devices with their personal smartphones. They also provided written instructions on how to use the digital technologies that participants took home and answered any questions about the technologies.

**Table 1.** Overview of all the digital technologies’ metrics and duration of use.

Technology type	Method of assessment	Metrics evaluated	Frequency of data collection	Level of engagement required from participants
Active smartphone app 1	Questionnaires and games	Speech Walking, gait, and balance Memory Reaction time	Once every 3 months	30‐35 minutes using the app
Active smartphone app 2	Questionnaires and games	Memory Attention Mental flexibility Speed of processing	Once every 3 months	10‐15 minutes using the app
Active smartphone app 3	Questionnaires and games	Memory Executive function Mood and sleep Subjective cognitive performance rating Speech	One task each day for 2 weeks Two-week assessment period reoccurs every 3 months	10‐15 minutes of using the app
Passive smartphone app	Speed and pattern of typing on the phone	Fine motor function	Turn on for data collection once every 3 months for 2 weeks	Data collected passively for 2 weeks without user input
Passive computer software	Speed and pattern of typing on the phone	Fine motor function	Turn on for data collection once every 3 months for 2 weeks	Data collected passively for 2 weeks without user input
Wrist-worn activity monitor 1 (can be alternated with the other wrist-worn activity monitor if the participant opted to use both devices)	Inertial measurement unit	Level of general activity Step count Sleep activity	Repeat 2-week study period every 3 months	Wear for the duration of the 2-week study period (including overnight)
Wrist-worn activity monitor 2 (can be alternated with the other wrist-worn activity monitor if the participant opted to use both devices)	Inertial measurement unit	General activity Sleep	Repeat 2-week study period every 3 months	Wear for the duration of the 2-week study period (including overnight)
Headband	Electroencephalogram to measure brain activity Accelerometer	Movement during sleep	Repeat 2-week study period every 3 months	Wear overnight for a minimum of 3 consecutive nights, ideally a full 2 weeks
Ring (worn on the thumb or index finger)	Oximeter Heart rate monitor	Heart rate Oxygen in the bloodstream Sleep quality and duration	Repeat 2-week study period every 3 months	Wear overnight for a minimum of 2 consecutive nights, ideally a full 2 weeks

### Data Collection

Participants completed a quantitative demographic questionnaire to gather relevant data on their sociodemographic background (eg, date of birth and biological sex [assigned at birth]) and medical history (eg, self-reported clinical diagnosis of MCI). Researchers (ZP or SR) shared information about this qualitative study with all participants. Researchers used the information provided in the questionnaire to monitor diversity across interested individuals to ensure that our sample consisted of both those living with a clinical diagnosis of MCI and those without any clinically diagnosed cognitive decline. A subgroup of self-selecting participants were invited to a remote (Zoom Technologies, Inc, or by telephone) semistructured interview after their first 2 weeks of using the digital technologies, occurring between April 2022 and June 2023. Interviews were conducted by a member of the research team (ZP or SR) who was trained by an experienced qualitative researcher (SW). All interviews were audio-recorded using the videoconference software or a dictaphone. A flexible interview topic guide (Multimedia Appendix 1), which was extensively piloted with 7 individuals with varying degrees of cognitive impairment and 2 caregivers, was used to guide and structure the discussions [22]. The topic guide was designed to gather participants’ perspectives on the different digital technologies they had selected to use.

### Data Analysis

Audio recordings of the interviews were transcribed verbatim by a transcription company (Datagain) and checked for accuracy by the research team (RK). Transcripts were uploaded to NVivo (version 14.23.2; QSR International) to assist our inductive thematic framework approach, a form of codebook thematic analysis [23,24]. Inductive thematic analysis is the process of identifying recurring concepts to create themes without using any preconceptions or preexisting framework [23]. This allowed us to focus on the views, perspectives, and experiences of participants without being influenced by any preexisting theories, ensuring that our analysis is an accurate reflection of the data [23]. The framework approach involved following 5 stages, beginning with members of the research team (SW and EB) familiarizing themselves with the data by reading and rereading the transcripts, before generating initial codes [23,24]. Core concepts (a central concept that holds associated shared meaning patterns, ie, themes) were then formed by grouping codes to create an analytical framework, which was applied across the whole dataset. A framework matrix was used to chart the data, consisting of rows (cases), columns (codes), and “cells” of data to help summarize the large quantity of data collected. This framework approach allowed multiple coders (SW and EB) to analyze the data and enabled our multidisciplinary team, with professional backgrounds in neuroscience, pharmacy, population health sciences, digital health inequities, physiology, and psychology, to review the analysis and interpret the data to provide a richer understanding of participants’ views and experiences [23,24]. The rigidity of the framework approach also allowed us to explore specific project aims (to explore the perspectives of potential end users on the acceptability of using a range of digital technologies) [23,24]. The constant comparative method, involving the researchers (SW and EB) moving backward and forward between the data, comparing data, and evolving explanations, was used to further develop key themes [25]. Regular meetings with authors (SW, EB, SPS, ZP, SR, and CT) were held throughout the analysis to ensure that the themes were accurately reflected by the data and that data saturation (the stage where repetitive codes or themes are identified, and no new information or relationships between them emerge in new transcripts) [26] was reached before participant recruitment stopped. Several factors were considered to identify when thematic data saturation was achieved, such as the quality of the interviews (eg, length and depth of detail captured), number of interviews, and lack of occurrence of new information in interviews conducted after thematic data saturation was thought to be achieved [26-29].

### Researcher Characteristics

The interviewers (ZP and SR) were male research assistants at Boston University School of Medicine at the time of this study and did not disclose their background to participants. There were no prior relationships between the participants and the research team. The interviewers were trained by a member of the team (SW) who had a master’s in cognitive neuroscience and experience in interviewing older adults and those living with cognitive decline (eg, MCI and dementia). The researchers involved in the analysis (SW, EB, CT, and SPS) were experienced qualitative researchers at various career stages, with diverse academic backgrounds in pharmacy, neuroscience, and public health. This multidisciplinary approach ensured a comprehensive understanding of participants’ experiences and mitigated bias from each researcher’s interests.

### Rigor and Trustworthiness

Various strategies were used to ensure that rigor was upheld during this qualitative research. This included following the SRQR (Standards for Reporting Qualitative Research) [30], completing the COREQ (Consolidated Criteria for Reporting Qualitative Research) checklist (Checklist 1) [31], and ensuring trustworthiness by following guidance outlined by Nowell et al [32] to achieve credibility, transferability, dependability, and confirmability of the study. To address credibility, we piloted the topic guide to ensure that the questions could be easily understood by participants. Transferability was achieved by providing a detailed description of the methods, allowing those who sought to transfer the findings to their own research or settings to judge it on a case-by-case basis. To achieve dependability, we used audit trails to clearly document logical decisions so others can understand how and why decisions were made. According to Lincoln and Guba [33], confirmability is established when credibility, transferability, and dependability are achieved.

### Ethical Considerations

This study was approved by the institutional review board at Boston University Medical Center (IRB: H-40542). This study was conducted in accordance with the principles of the Declaration of Helsinki (2024). Written and verbal informed consent to participate in the study was obtained from all participants. Participants were not remunerated after taking part in the study. Written informed consent to publish the findings was obtained from relevant participants. Participants were fully informed of the anonymization process (eg, names were replaced with an identification number).

## Results

### Participant Characteristics

In total, 24 individuals participated in this qualitative study, of which 13 had a clinical diagnosis of MCI, and 11 had no cognitive impairment but were aged 65 years and older. Half were female (n=12), and the average age of the sample was 72 years (Table 2). The specific type and number of technologies used by each participant over the first 2-week period are shown in Table 2. Semistructured interviews lasted between 25 and 45 minutes.

**Table 2.** Participant demographics and devices used.

ID	Sex	Diagnosis	Digital technology used									Total number of technologies over the first 2-week period
			Apps					Wearables				
			Active app 1	Active app 2	Active app 3	Passive smartphone app	Passive computer software	Wrist-worn activity monitor 1	Wrist-worn activity monitor 2	Headband	Ring	
P1	Female	No cognitive impairment	✓	✓	✓	✓		✓		✓	✓	7
P2	Male	MCI^a^	✓	✓	✓	✓	✓	✓		✓	✓	8
P3	Male	No cognitive impairment	✓	✓	✓	✓		✓			✓	6
P4	Female	No cognitive impairment	✓	✓	✓			✓		✓	✓	6
P5	Female	No cognitive impairment	✓	✓	✓	✓		✓	✓	✓	✓	8
P6	Female	MCI	✓	✓	✓	✓		✓		✓	✓	7
P7	Male	No cognitive impairment	✓	✓	✓	✓	✓		✓		✓	7
P8	Female	No cognitive impairment	✓	✓	✓	✓		✓	✓	✓	✓	8
P9	Male	No cognitive impairment	✓	✓	✓	✓	✓	✓	✓	✓	✓	9
P10	Female	No cognitive impairment		✓	✓	✓			✓	✓	✓	6
P11	Female	No cognitive impairment	✓	✓	✓	✓		✓		✓	✓	7
P12	Male	No cognitive impairment	✓	✓	✓	✓		✓	✓	✓	✓	8
P13	Male	MCI	✓	✓	✓	✓		✓	✓	✓	✓	8
P14	Male	MCI	✓	✓	✓	✓	✓	✓			✓	7
P15	Male	MCI	✓	✓	✓	✓	✓					5
P16	Male	MCI	✓	✓	✓	✓	✓	✓			✓	7
P17	Female	MCI						✓			✓	2
P18	Female	MCI	✓			✓		✓			✓	4
P19	Female	MCI	✓	✓		✓		✓		✓	✓	6
P20	Male	MCI	✓	✓	✓	✓	✓	✓		✓	✓	8
P21	Female	No cognitive impairment	✓	✓	✓	✓		✓		✓	✓	7
P22	Male	MCI	✓	✓	✓	✓		✓		✓	✓	7
P23	Female	MCI	✓	✓		✓		✓			✓	5
P24	Male	MCI	✓	✓	✓	✓	✓	✓		✓	✓	8

a MCI: mild cognitive impairment.

**Figure 1.**
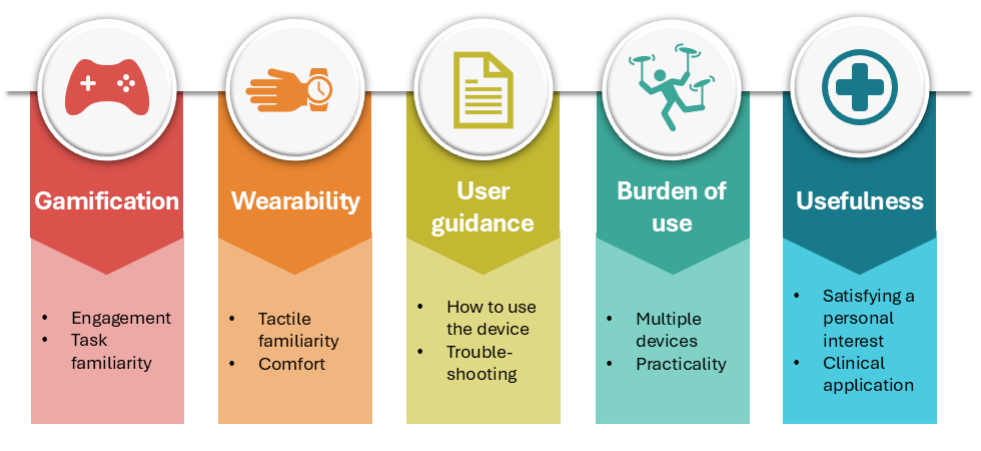
Summary of key themes and subthemes.

Our data analysis generated 5 key themes, which describe factors affecting the concurrent acceptability of a range of different digital technologies. These themes include (1) gamification, (2) wearability, (3) user guidance, (4) burden of use, and (5) usefulness (Figure 1). Each theme is described in detail below using direct anonymized quotes from participants; these appear in quotation markers (“”). To improve the flow and clarity, the author has added additional information to some quotes, indicated by square parentheses ([]), and removed some words, indicated by an ellipse in square parentheses ([…]). To ensure confidentiality, participant names have been replaced with their participant ID, which was assigned in chronological order, alongside additional relevant information to provide further context (eg, Participant 1, no cognitive impairment, used 7 technologies=participant who was interviewed first, did not have any diagnosed cognitive impairment, and used 7 of the 9 digital technologies available in this study). We take each of these themes in turn below.

### Gamification

Many participants living with MCI enjoyed the challenge of games within the active apps and felt that they were “keeping their brain alive” (Participant 23, living with MCI, used 5 technologies). Some participants without cognitive impairment who described themselves as competitive “wanted to get an A-Plus all the time” (Participant 8, no cognitive impairment, used 8 technologies). This participant described developing strategies in an attempt to improve their performance, such as verbalizing which direction they needed to swipe in the swiping task: “I think I did quite a bit better when I realized that saying it [the direction] out loud reinforced it” (Participant 8, no cognitive impairment, used 8 technologies). There was also a strong preference among those without cognitive impairment to select the active apps, which had “a little bit more variety” (Participant 7, no cognitive impairment, used 7 technologies) in the gamified tasks. This helped participants avoid the games becoming repetitive and boring. However, some older adults without cognitive impairment who had “never played video games” (Participant 4, no cognitive impairment, used 6 technologies) before felt that their lack of experience may have impacted their ability to complete the gamified task, as it took longer to understand the requirements: “I don’t play video games. So, it took me a while [to understand what to do]” (Participant 21, no cognitive impairment, used 7 technologies). When participants did not perform as well as they would have liked on the gamified tasks, they described feeling anxious at the thought of having to play the game again, reducing their motivation to use the app again.

### Wearability

Independent of cognitive decline, participants found the smart ring “easy [to use and] no big deal [to wear]” (Participant 11, no cognitive impairment, used 7 technologies). Similarly, most participants found the wrist-worn activity monitors acceptable because they were “just like wearing a watch, you know I don’t even know it’s there, except for the buzzes. So that’s fine” (Participant 4, no cognitive impairment, used 6 technologies). However, those who did not usually wear a watch discussed feeling the need to get “back in that habit” (Participant 1, no cognitive impairment, used 7 technologies), as they were “just not used to it” (Participant 9, no cognitive impairment, used 9 technologies). Many participants expressed hesitation around putting on the headband, as “it’s not the normal thing that I would like to be doing to go to sleep” (Participant 9, no cognitive impairment, used 9 technologies). Those who had used a medical wearable device on their head prior to this study were particularly concerned about the comfort of wearing it:

I had tried a sleep apnoea mask before, and I couldn’t do that [sleep with the mask on], and I didn’t like it. I thought this [the headband] was going to be similar.

[Participant 22, living with MCI, used 7 technologies]

In terms of comfort, some participants did not like the plastic straps on the wrist-worn activity monitor because “occasionally it got a little bit uncomfortable and sweaty, and if it’s too tight, it’s hard to move on my wrist” (Participant 5, no cognitive impairment, used 8 technologies). The headband also had some issues, as the electroencephalogram sensor prongs were felt to be “digging into the back of my head” (Participant 12, no cognitive impairment, used 8 technologies), causing discomfort and waking up participants during the night. This discomfort may have been linked to how tight the headband straps were adjusted, as one participant described how “the first night was terrible, but then I loosened [the straps], I used the extenders, so it wasn’t so tight and then it wasn’t quite so bad” (Participant 4, no cognitive impairment, used 6 technologies).

### User Guidance

All participants felt that the user guides provided by the research team were “very helpful” (Participant 1, no cognitive impairment, used 7 devices) and “easy to follow” (Participant 3, no cognitive impairment, used 6 technologies). However, the guidance given within the active and passive apps as well as the apps associated with the wearable technologies (used to collect data) required further improvement to support those with MCI. One participant described having “trouble with the [headband] around my [her] head because the directions were not clear enough” (Participant 19, living with MCI, used 6 technologies). In this case, the participant had worn the headband the wrong way around, and the data were not recorded over multiple nights. Others explained how it was not obvious “when it [wrist-worn device] needed to be charged” (Participant 8, no cognitive impairment, used 8 technologies) and would like more guidance on this.

A key area omitted in the user guides was how to troubleshoot technical issues. One of the most frequently reported technical issues involved difficulty syncing the headband and its associated app. Some participants described receiving “a pop up on the app that said [...], now that you’ve paired, the headband will recognize the device, but it never did” (Participant 5, no cognitive impairment, used 8 technologies). Another participant recalled how the smartphone app often froze, resulting in him having to “hit the [app home] button again” (Participant 14, living with MCI, used 7 technologies) to close the app, reopen it, and repeat the games. He described feeling frustrated at being unable to complete the required task in one go and questioned whether he was “doing something [wrong like] touching that is causing this or is it something in the game itself that is too sensitive?” (Participant 14, living with MCI, used 7 technologies). Experiencing such technical issues without any guidance on how to overcome them left some participants feeling “helplessness when it [the technology] doesn’t work” (Participant 24, living with MCI, used 8 technologies).

### Burden of Use

Participants with and without MCI described the burden of using 5 or more technologies, explaining how it was “a little invasive in terms of my time” (Participant 9, no cognitive impairment, used 9 technologies) and how they would have liked a shorter schedule of assessments so that it “would not wear me down” (Participant 21, no cognitive impairment, used 7 technologies). The burden of using the devices was particularly evident among participants who had “a lot going on in your [their] life” (Participant 6, living with MCI, used 7 technologies) or were on holiday during the 2-week period. One participant explained how they did not use the devices during their holiday as they “didn’t really want to have to do a lot of setting [up of the technology]” (Participant 11, no cognitive impairment, used 7 technologies). Some participants developed strategies to help “settle into some kind of pattern that was reasonable” (Participant 1, no cognitive impairment, used 7 technologies), with one participant making “a little chart of how many days I had to do this, that, and the next thing” (Participant 11, no cognitive impairment, used 7 technologies). This helped integrate the technology into their everyday lives.

Once a routine had been established, participants without cognitive impairment reflected on the practicality of using the devices. Some female participants were self-conscious of how the wearable devices looked on them, with one participant explaining how “if I’m going to wear anything on my wrist, if I wanted to, [it] would be a pretty bracelet” (Participant 11, no cognitive impairment, used 7 technologies). Other participants, who liked water sports, described the wrist-worn activity monitors as "a bit of a hassle [...] I was out in the swimming pool every day because I had to take the [monitors] off” (Participant 12, no cognitive impairment, used 8 technologies). As the devices were only water-resistant, not waterproof, some participants worried that they may have accidentally damaged the devices when they exposed them to water. One participant recounted how they

hopped into the shower with it. And I immediately hopped out and wiped it, and everything. It was fine. But I was a little bit, “Oh God, what have I done now?”

[Participant 11, no cognitive impairment, used 7 technologies]

### Usefulness

Participants found the digital technologies useful, particularly devices that allowed their scores on the gamified tasks to be compared over different days. Others living with a clinical diagnosis of MCI found that the health-related metrics (eg, heart rate) measured by the wrist-worn activity monitor were particularly useful. However, some participants living with MCI would have liked to interpret the feedback from the devices in further detail, particularly the sleep metrics: “I love looking at the feedback [sleep data from the headband] and, I wish I knew more about that so that was interesting” (Participant 6, living with MCI, used 7 technologies). Another participant without cognitive impairment felt that it would be useful to compare his metrics with others of a similar age:

It would be nice to be able to know that against this group of people [of similar age], you’re doing good, [or] you’re not doing good. So, if you need to change something [to improve your health and wellbeing], you could change it.

[Participant 7, no cognitive impairment, 7 technologies]

Most participants were hopeful that these digital technologies might be able to send reports directly to their health care provider. Others were more cautious and stated that they would “hate to see everything done high tech. I don’t want to lose the personal interaction between physicians and health care people and patients” (Participant 10, no cognitive impairment, used 6 technologies). Participants with existing health conditions worried about whether these conditions might impact the validity of the data collected by the digital technologies, with one explaining how she had a recent fall and “I think that they didn’t expect me to live, obviously I’ve done very well [with their health after the fall], but yes, I don’t think I’m as sharp [cognitively]” (Participant 10, no cognitive impairment, used 6 technologies). Some also questioned whether the data collected might be affected by treatment for a particular condition. For example, one participant who wore a sleep apnea mask at night queried how the oximeter sensor in the ring would “take into consideration that I’ve got oxygen on at night?” (Participant 10, no cognitive impairment, used 6 technologies).

## Discussion

### Principal Findings

This qualitative study explored the views of individuals potentially at risk of developing dementia, specifically older adults and those living with a clinical diagnosis of MCI, on the acceptability of using a range of different digital technologies. A range of 9 diverse technologies were used, and novel insights gained into the burden of using multiple devices and the key factors influencing acceptability. Most participants liked the gamified tasks in active apps, though some participants who were less familiar with digital games needed more time to adjust. Wearables placed on the body in areas of everyday accessories (eg, watches and rings) were preferred, but the straps used to fix the wearable to the body were sometimes found to be uncomfortable, particularly when worn too tightly. Clear, concise user instructions and troubleshooting guidance were found to be essential to ensure the proper use of the digital technologies, and further improvements were highlighted. Devices providing personally relevant feedback (eg, test scores or health metrics) were also well-received, although many questioned their usefulness within health care.

Gamification has great potential to increase participant engagement in cognitive tasks [34]. Findings from this study strongly support this stance, as gamification of cognitive testing through active smartphone apps was generally accepted by participants as challenging but also an engaging experience. Furthermore, a study conducted by Ríos Rincón et al [35] found that older adults preferred smartphone cognitive games compared to paper-based cognitive activities, and they experienced higher levels of engagement and positive cognitive effects while playing the smartphone games compared to paper-based activities. However, there are a number of considerations when using gamification in this manner. First, it is important to find the right balance between challenging the user with games, but also the goals within the games being achievable. Previous research has highlighted how if a digital game is too hard, users are more likely to experience low self-esteem, annoyance, and a sense of insecurity [36]. Second, an individual’s digital literacy skills, ability to use a hand-held digital device, and digital self-efficacy must be considered to avoid contributing toward digital health inequities once the technology is implemented into health care. This aspect is particularly important because individuals in this study were eligible to participate if they had owned a smartphone and had access to the internet at home. Some older adults have little to no experience of using games on a digital device, which they perceived had negatively impacted their ability to complete the task. The wider literature also suggests that older adults (aged 65 years and older) and those living with MCI are at risk of experiencing digital exclusion [37,38].

Where the device was worn on the body and the comfort of wearing the technology were 2 key elements found to affect the acceptability of a wearable device. Wrist-worn activity monitors and the smart ring were found to be more acceptable than the headband, which was consistent with previous findings [39-41]. This may be due to the increased familiarity and understanding of smartwatches [42,43] and the social acceptability of wearing the device on your wrist and using your hands to make gestures and touches to the device [39]. Comfort of the wearable devices when worn correctly versus incorrectly (eg, too tight) was also found in this study to impact acceptance, which is echoed in the wider literature [44,45]. This highlights the importance of providing adequate and clear user instructions on how to wear and use the digital technologies.

User instructions must also contain guidance on how to overcome technical issues, such as how issues with syncing a wearable device to its associated smartphone app and what to do if an active smartphone app freezes. These instructions need to be written in such a way that they are suitable for those with cognitive impairment. Experiencing technical issues without any guidance on how to overcome them led to some participants in this study feeling hopeless. Law et al [46] found that professional caregivers of those with cognitive decline (health care professionals and staff at a retirement facility) were concerned that technical issues with digital technologies would cause confusion among those with cognitive impairments and would likely prevent them from engaging with the technology if the issues could not be fixed. Therefore, technical support must be given to end users and caregivers of those with cognitive impairment to promote acceptance, use, and engagement with the devices.

The burden placed on end users when using multiple devices was another key area that emerged in this study. Those who used 5 or more different digital technologies felt a high level of burden, and some found it challenging to integrate these devices into their busy daily lives. However, using digital technologies to support the early detection of dementia-causing diseases is viewed as a less invasive approach, but we found that the greater the number of devices that a participant used, the more it can potentially be invasive of their time. Furthermore, it brings into question if digital technology’s ability to offer remote data collection and potentially shorter assessment periods only displaced the burden from health care systems onto end users rather than reduced the burden overall [47]. To reduce end-user burden, this study suggests that users should not be asked to use more than 4 devices at any 1 time and that the devices are waterproof to reduce the number of times the device needs to be removed. The wider literature also suggests selecting digital technologies that are easy to use and/or wear and devices that need minimal interaction (eg, have a long battery life to reduce charging) to reduce the number of tasks required [47,48].

The ability of the devices to provide personal feedback, such as test scores and health-related metrics (eg, heart rate), was perceived as useful and interesting by participants in this study. Such a finding is echoed in the literature, which also suggests that feedback from digital health technologies drives engagement and continued use of technologies among older adults [44,48]. Previous studies have also found that feedback from activity monitors may motivate older adults to increase their physical activity [39]. Despite the benefits of digital technologies in satisfying personal interests, many participants also raised concerns about potentially losing valued personal interactions with health care professionals and whether their existing health conditions and treatment may affect the validity of the data collected. Similar concerns about losing human connection in health care interaction have been reported in the wider literature [49]; however, there has been little to no research at the time of this study, discussing users’ concerns about how their health conditions and medical treatments may affect the validity of the data. This novel insight must be further explored to understand to what extent health conditions and treatments are likely to impact the validity of the data collected and how to mitigate such concerns.

### Strengths, Limitations, and Areas for Future Research

Multiple strategies were used in this study to ensure rigor and trustworthiness, such as collecting data until thematic data saturation was achieved and piloting the topic guide [31,32]. The inductive nature of our analysis supported the identification of novel insights, such as the burden of using multiple devices and concerns regarding the impact of existing health conditions on specific metrics. Furthermore, the range of different technologies used in the study provides originality and contributes to our understanding of accessibility and usability of technologies for health care purposes. A key limitation of this study is that individuals self-selected to participate in the study and were required to own a smartphone and have access to internet connectivity at home. There were no strategies put in place to support the recruitment of those who may have experienced or are experiencing digital exclusion, such as those of low socioeconomic status who cannot afford a smartphone, or those who live in rural areas who may not have access to a reliable internet connection at home [37]. Some insights into those with little to no experience of using digital technologies (eg, games on a smartphone via an app) were gathered in this study, but further research is needed to gain a deeper understanding of the unique views, perceptions, and opinions of those experiencing digital exclusion and to ensure that the digital intervention does not contribute to digital health inequalities when implemented in practice. Although our study provides a rich in-depth understanding regarding acceptability, usability, and user burden, the inclusion of a quantitative measure of acceptability, usability, and/or burden, such as System Usability Scale [50], could have facilitated data triangulation and potential comparisons to be made between technologies and subgroups (MCI vs no impairment or high vs low device count).

### Conclusions

This novel qualitative study highlighted factors affecting concurrent acceptability of using a range of digital technologies that have the potential to support early dementia detection from the perspectives of potential end users. This information will help contribute to researchers’ decision-making regarding how many devices the participants should be asked to use and the key elements (eg, comfort, place a device is worn, and waterproof) of a digital device that must be considered to improve acceptance and engagement. Future research is needed to gain a deeper understanding of the factors that affect the acceptance of using digital technologies to support early dementia detection among those experiencing digital exclusion, as our recruitment design could be viewed as limiting inclusion of this population, and their views are necessary to ensure that digital health equity can be supported.

## Supplementary material

10.2196/84004 Multimedia Appendix 1 Interview topic guide.

10.2196/84004 Checklist 1 COREQ checklist.

## Abbreviations

BU-ADRC: Boston University-Alzheimer’s Disease Research Centre
COREQ: Consolidated Criteria for Reporting Qualitative Research
EDoN: Early Detection of Neurodegenerative Diseases
MCI: mild cognitive impairment
SRQR: Standards for Reporting Qualitative Research
