# Supplementary material for: Acceptability of Technologies to Support Early Dementia Detection: Qualitative Study With the Boston University Alzheimer’s Disease Center Cohort
Source: J Med Internet Res. 2026 May 29;28:e84004. doi: 10.2196/84004 (PMC13220977; doi:10.2196/84004)
Supplement: Multimedia Appendix 1 [file jmir-v28-e84004-s001.docx]

**PPI Interview Topic Guide**

I would like to start by thanking you for making time to take part in today’s interview. I am interested in understanding your experience of the wearable technology we sent you and the apps we asked you to install.  This could be used to aid our research project, which is looking into ways to detect conditions such as dementia earlier than is currently possible.

Before we start the interview, I would like to ask you if it is okay to record our conversation?

**Cognitive impairment assessment**

- Have you been diagnosed with dementia or mild cognitive impairment?

*Prompts after the question is asked to gather more info:*

*do you know the approximate month and year this diagnosis was given*?

*How do you think your memory and thinking has been since diagnosis?*

- Are you able to do everyday activities by yourself such has taking care of yourself, making a cup of coffee and going shopping?

We will now ask a few questions about the devices you have been using…

1. What devices have you been using?
2. What has your experience been like with the devices over the past 2 weeks?

*Did it go as expected? Has anything surprised you?*

1. Did you opt in or opt out any devices?

*Which devices did you opt in/out?*

*When and why was this?*

*What do you think could have motivated you to keep using the device?*

1. What are your opinions on the devices now that you’ve used them for a while?

*e.g., Perceived ease of use, benefits of using the system, perceived value or potential of these tools for understanding their own brain health and ageing?*

*Is there any particular devices that you prefer*?

*If the devices gave cross over data (i*.e*. fitbit and dreem give sleep data) did you look at the comparisons between the data it shown*

1. Do you think your behaviours or health has changed as a result of using the device over the course of the last 2 weeks?

*particularly in the case of devices like a wrist worn activity monitor which tells you information about your steps, heart rate etc.*

1. Were any features troublesome or inconvenient with the wearables?

*Font size, comfort? Colour, size (aspects of the apps/ devices that can be customised?)*

*Suggestions for improvement?*

1. Did you use anything to help you use the devices?

*Was the guidance provided useful?*

*Is there any additional support you think would be beneficial?*

1. Do you have any concerns about any risks of using the device?

*Particularly around data security (if they used longevity specifically ask about this as issues were raised before)*

*If so what made you feel concerned? (if you can try to tell them how their data is safe)*

1. Do you feel that you have any health conditions that affected your use of the wearables?

*issues with holding the devices, typing on your phone or pressing buttons on any of the devices.*

1. If these devices were part of a standard health check that are normally provided for certain age categories would you be willing to use them?

*(if they say all devices would be too much ask how many they would be willing to use and which ones)*

1. Do you think you will carry on using the devices over 2 years?
2. Is there any other feedback you’d like to provide?
